# COVID-19: Perceived Infection Risk and Barriers to Uptake of Pfizer-BioNTech and Moderna Vaccines Among Community Healthcare Workers

**DOI:** 10.1007/s40615-021-01093-6

**Published:** 2021-07-15

**Authors:** Tolulope B. Famuyiro, Abayomi Ogunwale, Jude des Bordes, Mukaila Raji

**Affiliations:** 1 Department of Family and Community Medicine, The University of Texas McGovern Medical School, Houston, TX USA; 2 Division of Geriatrics and Palliative Medicine Department of Internal Medicine Department of Preventive Medicine and Population Health, University of Texas Medical Branch, Galveston, TX USA

**Keywords:** COVID-19 vaccine, Vaccine uptake, Vaccine hesitance, Healthcare workers, COVID-19

## Abstract

**Background:**

The health and economic ramifications of the coronavirus pandemic have prompted the need for a timely and effective vaccine development. While the rollout of the COVID-19 vaccine in record time is being hailed as a scientific feat, skepticism about the safety, side effects, and even its long-term effects remain. Acceptance of the vaccine may therefore be a challenge among healthcare workers (HCWs), whose role is considered a proxy to determining the COVID-19 vaccine uptake response by the general population.

**Methods:**

In December 2020, prior to the arrival and receipt of the Pfizer-BioNTech and Moderna COVID-19 vaccine, we conducted a cross-sectional survey to assess the readiness for vaccine uptake among HCWs at three community-based, university-affiliated health centers.

**Results:**

A total of 205 (82%) respondents out of 250 completed the questionnaire. Fifty-four percent of respondents agreed to receive vaccine once available. Females (odds ratio (OR) =0.22, p=0.014), non-Hispanic Blacks (OR=0.066, p=0.010), and Hispanics (OR=0.11, p=0.037) were less likely to accept the vaccine. Respondents with moderate-risk perception were more likely to accept (OR=2.79, p=0.045) compared to those with low-risk perception while no association was found between high-risk perception and vaccine acceptance (p=0.226). After adjusting for perceived risk, sex, race/ethnicity, and age, acceptance in non-Hispanic Black population remained statistically significant (adjusted OR=0.07, p=0.014), with Hispanic (AOR=0.12, p=0.051) showing a trend.

**Conclusions:**

Enthusiastic acceptance of the COVID-19 vaccine is lacking among surveyed HCWs of certain racial/ethnic groups. Provision of resources and public health interventions targeting underserved, minority populations are necessary to halt opposition to vaccine uptake.

## Introduction

In November 2019, severe acute respiratory disease coronavirus 2 (SARS-CoV-2) emerged from Wuhan, China, and rapidly spread creating a pandemic known as coronavirus (COVID-19) infection. Across the world, the USA records the highest confirmed cases and death [1–4]. The age-specific mortality report published in the *Journal of the American Medical Association* lists COVID-19 infection as one of the top leading causes of death for persons aged 45 and older in the USA [5]. Nationally, the COVID-19 pandemic disproportionately harms minorities, with American Indian, African American, and Hispanic communities reporting more hospitalizations and deaths [4].

The health and economic ramifications of the pandemic prompted the need for a timely and effective vaccine development. Less than a year after the first COVID-19 case was identified, two mRNA-based vaccines by Pfizer-BioNTech and Moderna were developed [2, 6]. Following the release of the phase 3 clinical study trial report showing high antibody response, as well as immunogenicity among its recipients, the US Food and Drug Administration granted these two vaccines emergency use authorization for active immunization in the USA [2, 7–12]. While the rollout of the COVID-19 vaccine in record time is being hailed as a scientific feat, skepticism about the safety, side effects, and its long-term effects remains. Concerns about politicization of the process have also been expressed, among others. In light of these skepticisms, acceptance of the COVID-19 vaccine by those eligible for early vaccination could pose a threat to achieving much-needed national herd immunity [2, 8–10, 12–18].

The vaccine rollout strategy recognized and prioritized healthcare workers to be the first to receive the vaccine, a decision based on the premise that HCWs serving in the frontline of defense against this infection were most at risk of getting and transmitting the virus to others [2, 8, 11, 13–17, 19, 20]. With reports from older studies showing a low uptake of influenza vaccine among HCWs during the H1N1 pandemic in 2009 and the knowledge of how vital the role healthcare workers (HCWs) play in determining the response of the general population towards vaccine acceptance, this study assessed the perceived acceptance readiness of community healthcare workers to the Pfizer-BioNTech and Moderna vaccines and to identify factors that influence their decisions [21, 22]. We hypothesized that perception of increased risk for COVID-19 infection strongly influences the decision of HCWs to receive COVID-19 vaccine and that HCWs involved in direct patient care are more likely to agree to receive COVID-19 vaccine immediately rather than later compared to nonclinical staff. Further, that vaccine safety is the most likely concern influencing vaccine uptake decision.

## Methods

A cross-sectional study was conducted in December 2020 (prior to the arrival and receipt of the Pfizer-BioNTech and Moderna vaccines). A survey was created and individually disseminated among the community clinical and nonclinical HCWs of three community-based, university-affiliated health centers in Houston, Texas. The study was approved by the Institutional Review Board of the University of Texas Health Science Center, Houston. Demographic information collected include age, sex, race, ethnicity, and HCW roles (divided into four groups: attending physician, residents, other clinical staff involved in direct patient care (e.g., nurses, medical assistants, respiratory therapists), and others without direct patient care, such as receptionists, administrative personnel, and social workers). Exposure to COVID-19 was assessed by a question on whether respondents had contact (within a 6-ft distance) with a COVID-19 positive patient or a person under investigation (PUI) for the infection or if they had ever tested positive for COVID-19 infection. Perceived infection risk for COVID-19 was assessed with questions **“**How do you perceive your risk of getting COVID-19 infection?” matched with responses: low, moderate, or high risk. The drivers to the decision to receive the vaccine were obtained with a question asking whether the respondents’ perceived risk of getting COVID-19 influenced their decision to receive or not receive the vaccine. In addition, we asked if the decision to receive the vaccine was driven by other factors such as their personal medical history (such as known history of diabetes, underlying lung, or heart disease), work exposure risk, recommendation by the employer, and data from a public health expert, media, family, or colleague. Vaccine uptake readiness (outcome) was assessed with responses: “I would like to receive the vaccine once available,” “I would like to receive the vaccine but prefer to wait until later,” or “I do not plan on receiving the vaccine.” Also, we gauged their willingness to recommend the vaccine to others on a Likert scale with the question: “How likely are you to recommend the vaccine to others?” Finally, barriers and concerns relating to the COVID-19 vaccine were assessed with a question identifying the issue(s) most concerning to them about the recently approved COVID-19 vaccine.

Overall data were reported using the frequentist inference while associations between outcomes and the independent variables were analyzed using logistic regression.

## Results

A total of 205 (82%) HCWs out of 250 completed the questionnaire. Respondents were mostly young HCWs ages 25–34 years (42%, n=86), females (75%, n=152), and Non-Hispanic Blacks (32%, n=64). Most respondents were clinical staff directly involved in patient care (86%, n=178). Over half of the total respondents (54%) agreed to receive vaccine once they become available while the rest would either wait (27.7%) or not receive the vaccine at all (17.8%)**.** Table 1 is a summary of the respondents’ characteristics and vaccine uptake willingness.

**Table 1:** Respondents’ characteristics matched to their vaccine uptake readiness response

	Willingness to accept vaccine, N (%)			Total, N
Characteristic	No (N=36)	Yes (once available) N=110	Yes (later) N=56	
Age group				
18–24	3 (33.3)	3 (33.3)	3 (33.3)	9
25–34	11 (12.9)	50 (58.8)	24 (28.2)	85
35–44	7 (16.3)	25 (58.1)	11 (25.6)	43
45–54	11 (28.2)	16 (41.0)	12 (30.8)	39
55–64	4 (21.0)	10 (52.6)	5 (26.3)	19
≥65	0 (0.0)	6 (85.7)	1 (14.3)	7
Sex				
Female	33 (22.2)	67 (45.0)	49 (32.9)	149
Male	3 (5.8)	43 (82.7)	6 (11.5)	52
Race/ethnicity				
Non-Hispanic White	1 (3.1)	23 (71.2)	8 (25.0)	32
Non-Hispanic Black	21 (32.8)	24 (37.5)	19 (29.7)	64
Hispanic	13 (22.8)	26 (45.6)	18 (31.6)	57
Asian	0 (0.0)	32 (78.1)	9 (22.0)	41
Other	1 (25.0)	2 (50.0)	1 (25.0)	4
Direct patient care				
Yes	29 (16.6)	97 (55.4)	49 (28.0)	175
No	6 (26.1)	10 (43.5)	7 (30.4)	23
Perceived risk				
Low	8 (30.8)	11 (42.3)	7 (26.9)	26
Moderate	14 (13.7)	57 (55.9)	31 (30.4)	102
High	14 (19.2)	41 (56.2)	18 (24.7)	73
COVID-19 patient contact				
Yes	17 (13.3)	74 (57.8)	37 (28.9)	128
No	19 (26.4)	34 (47.2)	19 (26.4)	72
Position (clinical role)				
Physician/attending	0 (0.00)	25 (83.3)	5 (16.7)	30
Resident	3 (5.6)	44 (81.5)	7 (13.0)	54
Other clinical staff	26 (28.6)	28 (30.8)	37 (40.7)	91
Non-clinical staff	6 (26.1)	10 (43.5)	7 (30.4)	23

**Fig. 1:**
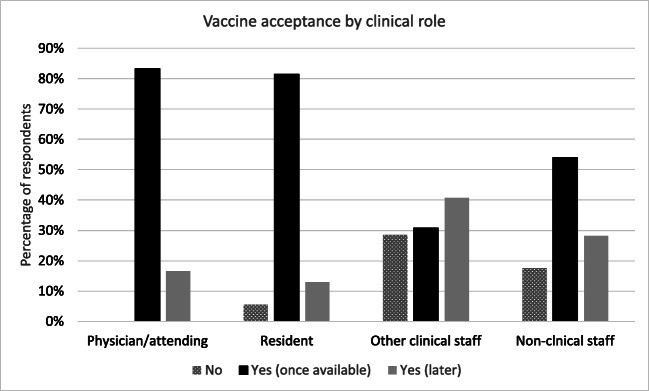
Vaccine acceptance by clinical role

**Fig. 2:**
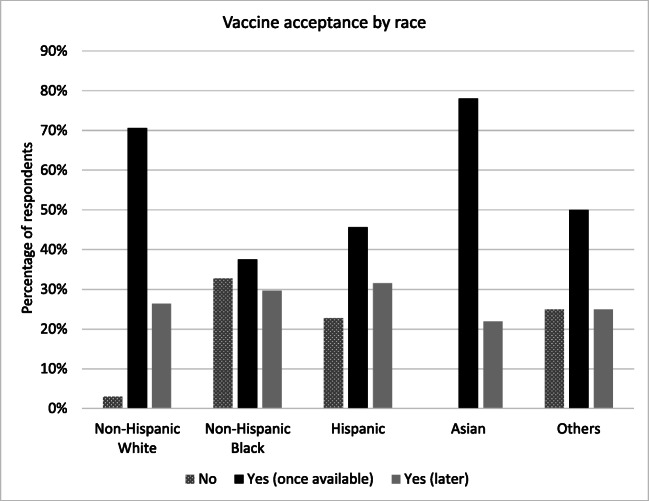
Vaccine acceptance by race/ethnicity

**Fig. 3:**
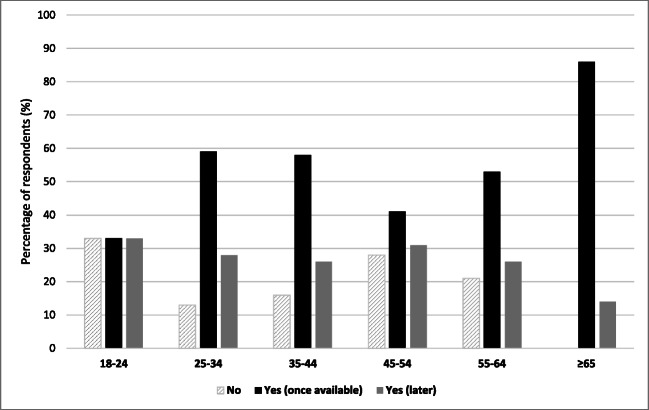
Vaccine acceptance by age of respondents

Most physicians (83%) and residents (81%) expressed more enthusiasm to receive the vaccine once it became available compared to other clinical staff (nurses, medical assistant, clinical technician, etc.) (31%) (Figure 1), while 78% of Asians and 71% of non-Hispanic whites were willing to receive the vaccine as soon as available 46% of Hispanics and 36% of non-Hispanic blacks were willing to do same (Figure 2). Respondents 65 years and older were most willing to immediately receive the vaccine (86%) compared to all other age groups (Figure 3). Among those who were hesitant, NHB constituted 44% (n=40), Hispanics 34% (n=31) and 10% (n=9) each for non-Hispanic Whites and Asians.

**Fig. 4:**
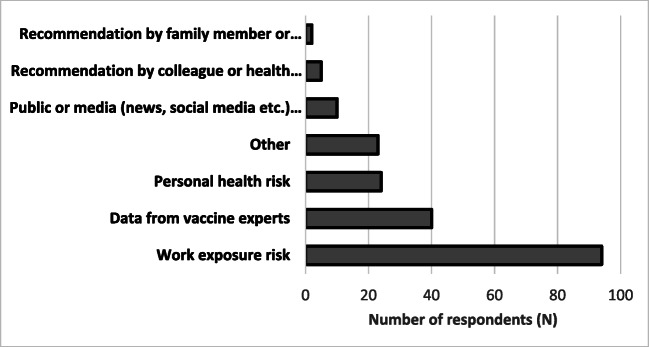
Factors influencing COVID-19 vaccine uptake decision

Work exposure risk (47%, n=94) and data from experts (20%, n=40) most influenced HCWs’ vaccine uptake decision (Figure 4), while vaccine safety (80%, n=161) and efficacy (13%, n=20) served as potential barriers to uptake. Sixty-seven percent of all respondents would recommend the vaccine to friends and family.

Bivariate analyses showed that females (compared to males) were less likely to agree to receive the vaccine (OR=0.22, P=0.014). Non-Hispanic Blacks (OR=0.066, p=0.010) and Hispanics (OR=0.11, p=0.037) were less likely to accept the vaccine compared to non-Hispanic Whites. Overall, the perceived risk of getting COVID-19 infection did not influence vaccine acceptance. However, respondents with moderate-risk perception were more likely to accept (OR=2.79, p=0.045) compared to those with low-risk perception while no association existed between high-risk perception and vaccine acceptance (p=0.226). On adjusting for perceived risk, sex, race/ethnicity, and age, only non-Hispanic Black remained statistically significant (adjusted OR=0.07, 95% confidence interval (CI), 0.01–0.59), while moderate-risk perception (adjusted OR=3.17, p=0.055) and Hispanic (adjusted OR=0.12, p=0.051) showed a trend towards significance. There was no significant association between vaccine uptake response and whether or not HCW provided direct care.

## Discussions

Almost half of our study respondents (45.5%) would either not receive the Pfizer-BioNTech or Moderna vaccines at all, or not receive it immediately. This points to a high level of vaccine hesitancy in a critical group in the vaccine supply chain which, if left unaddressed, may amplify the opposition to the vaccine that may already exist in many communities. The lack of enthusiasm towards the COVID-19 vaccine which has been widely reported in the public appears to be reflective in the attitude and perception of HCWs. It is particularly concerning that the subgroups—Blacks and Hispanics—with highest rate of vaccine non-acceptance in our study also carry the highest rate of infection, hospitalization, and death from COVID-19 [23]. It was not surprising that there was a high degree of hesitancy among “other clinical staff” who were mainly (82%) non-Hispanic Black and Hispanics.

Reluctance to receive these vaccines may be driven by doubts about immediate and long-term safety and efficacy, lack of trust in the development process, as well as background historical vaccine apathy in minority populations. Hence, the provision of educational resources to equip HCWs about the safety, importance of vaccination, and negative implication of refusing the vaccine should be implemented.

As timing may be crucial to the success of widespread vaccination, the release of vaccine adverse event report (VAER) should not be delayed as this may retard the progression of the decision of those respondents who opted to wait to receive the vaccine to wanting the vaccine earlier. So far, the CDC VAER shows only a few side effects ranging from headaches, vaccine injection site pain, mild to moderate anaphylactic reactions, and death (n=1000 deaths, 0.003%) that were not directly linked to the vaccine [18, 24]. As more postvaccination reports reveal non-life-threatening adverse events, a decrease rate of incidence, prevalence, hospitalization, as well as morbidity and mortality rates of COVID-19 infection, those who have previously expressed hesitancy may begin to reconsider their decisions to receive the vaccine.

Non-Hispanic Blacks (and probably Hispanic and female HCWs) were more hesitant towards receiving the COVID-19 vaccine, a finding which is consistent with prior studies that assessed perception and attitude of HCWs in large tertiary hospitals [11, 17]. This report calls for a need to identify and establish a vaccine champion coalition consisting largely of minority HCWs who can act as a resource and encouragement to their vaccine-hesitant colleagues. Another potential approach to mitigating COVID vaccine hesitancy among racial and ethnic minorities is enlisting the support of community stakeholders (e.g., religious leaders and others in faith-based organizations) who have been shown to hold substantial influence in communities of color [25]. These community influencers might likely be able to understand how social determinants of health (e.g., suboptimal housing, transportation, and health insurance) might impact vaccine-related decision-making and what approaches to recommend promoting vaccine acceptance in the community of colors. Finally, there is a need for more active roles by local business leaders in their communities not just in encouraging and educating their employees to accept COVID vaccines but to provide human and capital resources (e.g., time off, transportation) to facilitate the process of their employees getting the COVID vaccines when they become available (US Department of Health and Human Services, January 2021) [26].

Regarding the perception of infection risk, respondents with moderate-risk perception who affirmed to getting the vaccine once available were mostly the millennials and likely fall into the category of the presumed healthy population. These respondents perceived risk relied solely on work exposure vulnerability, rather than on personal medical history.

Despite their personal decision to receive or not receive the vaccine, most respondents still strongly agreed to recommend the COVID-19 vaccine to others. As such, utilizing HCWs to advocate for early COVID-19 vaccination among the public seems like a prudent approach. Additionally, the convenience of receiving two doses of vaccine was not identified as a barrier to uptake, although realistically, receiving one dose over two doses could eliminate production-delivery-administration hurdles and concomitantly increase vaccination coverage.

It must be noted that our sample was not reflective of the racial composition of the Texas healthcare workforce because we recruited willing participants. The possible selection bias of a convenient sample is a limitation; however, the diverse representation of respondents across all ages, gender, race, and ethnicity strengthens the result. The survey reliance on self-reported data and small sample size may have introduced Hawthorne bias and limit generalizability. However, the presence of similarities in findings between our report and prior studies conducted at a large tertiary institution is a strength [11]. While these results cannot fully explain the drivers of the initial hesitance of community healthcare workers towards the COVID-19 vaccine, our report certainly provides insight to inform strategies to promote effective vaccination distribution and administration efforts. Future studies on social determinants of health (e.g., perception of current job security as HCW, multigenerational housing involving living with vulnerable relatives—the elderly and persons on dialysis, perception of access to PPE at current workplace, quality of healthcare insurance, housing, vaccine-related religious belief) are also needed to further understand their roles as potential drivers of COVID vaccine hesitancy among healthcare workers. It would also be informative for the current participants in our study to be reinterviewed 6 months later with an expanded questionnaire to study, (a) concordance of pre-vaccine answers to the current period of vaccine availability by comparing their answers on willingness to accept the vaccine to whether they actually receive the vaccines which are now available and (b) rate of COVID-19 screening, infection, hospitalization and death, and relate the outcomes to prior answers on the perception of risk of being infected with coronavirus and willingness to accept COVID vaccination. Findings from such studies can inform the design and implementation of public health programs to improve COVID vaccine uptake and mitigate vaccine inertia especially in populations with the highest risk of severe COVID-19—the American Indian, African American, and Hispanic communities [4].

## Conclusions

Our report shows a racially disproportionate COVID-19 vaccine uptake perception exists among HCWs, particularly those of the non-Hispanic Blacks and Hispanic population. Despite widespread data and the health implication of these disease, enthusiastic acceptance of the COVID-19 vaccine is lacking among surveyed HCWs, in large part due to concerns regarding the safety and side effects. Upholding scientific data report on the efficacy of Pfizer-BioNTech and Moderna vaccine and launching vaccine champions consisting largely of non-Hispanic Black and Hispanic HCWs could help to rebuild the trust and restore confidence in the vaccine. Further, public health officials should seek to maintain transparency by expediting the public release of all findings relating to the vaccines.

## Data Availability

The data presented in this study are available on request from the corresponding author. The data are not publicly available due to participant and organizational privacy concerns.

## References

[1] World Health Organization (2020) WHO Coronavirus (COVID-19) Dashboard. https://covid19.who.int/. Accessed December 1, 2020

[2] Fisher KA, Bloomstone SJ, Walder J, Crawford S, Fouayzi H, Mazor KM (2020). Attitudes toward a potential SARS-CoV-2 vaccine: a survey of U.S. Adults. Ann Intern Med.

[3] JHU Coronavirus Resource Center (2020) COVID-19 Dashboard by the Center for Systems Science and Engineering (CSSE) at Johns Hopkins University . https://coronavirus.jhu.edu/map.html Accessed November 1, 2020

[4] The Covid Tracking Project (2020) The COVID racial data tracker. https://covidtracking.com/race Accessed November 11, 2020

[5] Woolf SH, Chapman DA, Lee JH (2021). COVID-19 as the leading cause of death in the United States. Jama.

[6] Amawi H, Abu Deiab GI, AA AA, Dua K, Tambuwala MM (2020). COVID-19 pandemic: an overview of epidemiology, pathogenesis, diagnostics and potential vaccines and therapeutics. Ther Deliv.

[7] Alturki SO, Alturki SO, Connors J, Cusimano G, Kutzler MA, Izmirly AM, Haddad EK (2020). The 2020 pandemic: current SARS-CoV-2 vaccine development. Front Immunol.

[8] Haynes BF (2021). A new vaccine to battle Covid-19. N Engl J Med.

[9] Jackson LA, Anderson EJ, Rouphael NG, Roberts PC, Makhene M, Coler RN, McCullough MP, Chappell JD, Denison MR, Stevens LJ, Pruijssers AJ, McDermott A, Flach B, Doria-Rose NA, Corbett KS, Morabito KM, O ' Dell S, Schmidt SD, Swanson PA, Padilla M, Mascola JR, Neuzil KM, Bennett H, Sun W, Peters E, Makowski M, Albert J, Cross K, Buchanan W, Pikaart-Tautges R, Ledgerwood JE, Graham BS, Beigel JH (2020). An mRNA vaccine against SARS-CoV-2 - preliminary Report. N Engl J Med.

[10] Lotfi M, Hamblin MR, Rezaei N (2020). COVID-19: transmission, prevention, and potential therapeutic opportunities. Clin Chim Acta.

[11] Shekhar R, Sheikh AB, Upadhyay S, Singh M, Kottewar S, Mir H, et al. COVID-19 Vaccine Acceptance among health care workers in the United States. Vaccines (Basel). 2021;9(2). 10.3390/vaccines9020119. 10.3390/vaccines9020119 PMC7913135 33546165

[12] Walsh EE, Frenck RW, Falsey AR, Kitchin N, Absalon J, Gurtman A, Lockhart S, Neuzil K, Mulligan MJ, Bailey R, Swanson KA, Li P, Koury K, Kalina W, Cooper D, Fontes-Garfias C, Shi PY, Türeci Ö, Tompkins KR, Lyke KE, Raabe V, Dormitzer PR, Jansen KU, Şahin U, Gruber WC (2020). Safety and Immunogenicity of Two RNA-Based Covid-19 vaccine candidates. N Engl J Med.

[13] Lin Y, Hu Z, Zhao Q, Alias H, Danaee M, Wong LP (2020). Understanding COVID-19 vaccine demand and hesitancy: a nationwide online survey in China. PLoS Negl Trop Dis.

[14] Malik AA, McFadden SM, Elharake J, Omer SB (2020). Determinants of COVID-19 vaccine acceptance in the US. EClinicalMedicine.

[15] Paterson P, Meurice F, Stanberry LR, Glismann S, Rosenthal SL, Larson HJ (2016). Vaccine hesitancy and healthcare providers. Vaccine.

[16] Verger P, Fressard L, Collange F, Gautier A, Jestin C, Launay O, Raude J, Pulcini C, Peretti-Watel P (2015). Vaccine hesitancy among general practitioners and its determinants during controversies: a national cross-sectional survey in France. EBioMedicine.

[17] Gagneux-Brunon A, Detoc M, Bruel S, Tardy B, Rozaire O, Frappe P, Botelho-Nevers E (2021). Intention to get vaccinations against COVID-19 in French healthcare workers during the first pandemic wave: a cross-sectional survey. J Hosp Infect.

[18] Shimabukuro TT, Cole M, Su JR (2021). Reports of anaphylaxis after receipt of mRNA COVID-19 vaccines in the US-December 14, 2020-January 18, 2021. Jama.

[19] Freeman D, Loe BS, Chadwick A, Vaccari C, Waite F, Rosebrock L, et al. COVID-19 vaccine hesitancy in the UK: the Oxford coronavirus explanations, attitudes, and narratives survey (Oceans) II. Psychol Med. 2020:1–15. 10.1017/s0033291720005188. 10.1017/S0033291720005188 PMC7804077 33305716

[20] Wong MCS, Wong ELY, Huang J, Cheung AWL, Law K, Chong MKC, Ng RWY, Lai CKC, Boon SS, Lau JTF, Chen Z, Chan PKS (2021). Acceptance of the COVID-19 vaccine based on the health belief model: a population-based survey in Hong Kong. Vaccine.

[21] MacDonald NE, Dubé E (2015). Unpacking vaccine hesitancy among healthcare providers. EBioMedicine.

[22] Zhang J, While AE, Norman IJ (2012). Seasonal influenza vaccination knowledge, risk perception, health beliefs and vaccination behaviours of nurses. Epidemiol Infect.

[23] Centers for Disease Control and Prevention (2021) Risk for COVID-19 infection, hospitalization, and death by race/ethnicity. https://www.cdc.gov/coronavirus/2019-ncov/covid-data/investigations-discovery/hospitalization-death-by-race-ethnicity.html. Accessed March 17, 2021

[24] Shimabukuro T (2021) COVID-19 Vaccine safety update, Advisory Committee on Immunization Practices (ACIP). https://www.cdc.gov/vaccines/acip/meetings/downloads/slides-2021-01/06-COVID-Shimabukuro.pdf. Accessed February 2, 2021

[25] Holden K, Akintobi T, Hopkins J, Belton A, McGregor B, Blanks S, et al. Community engaged leadership to advance health equity and build healthier communities. Soc Sci (Basel). 2016;5(1). 10.3390/socsci5010002. 10.3390/socsci5010002 PMC5048675 27713839

[26] U.S. Department of Health and Human Services (2021) Community health and economic prosperity: engaging businesses as stewards and stakeholders—a report of the surgeon general. . U.S. Department of Health and Human Services, Centers for Disease Control and Prevention, Office of the Associate Director for Policy and Strategy, Atlanta ,GA

